# Multi-parameter Behavioral Phenotyping of the MPP+ Model of Parkinson’s Disease in Zebrafish

**DOI:** 10.3389/fnbeh.2020.623924

**Published:** 2020-12-18

**Authors:** Christian Christensen, Haraldur Þorsteinsson, Valerie Helene Maier, Karl Ægir Karlsson

**Affiliations:** ^1^3Z Ehf, Reykjavik, Iceland; ^2^Biomedical Center, University of Iceland, Reykjavik, Iceland; ^3^Department of Engineering, School of Technology, Reykjavik University, Reykjavik, Iceland

**Keywords:** MPP+, zebrafish, Parkinson’s, behavior, GDNF, 4-phenylbutyrate

## Abstract

Parkinson’s disease (PD) has been modeled in several animal species using the neurotoxins 1-methyl-4-phenyl-1,2,3,6-tetrahydropyridine (MPTP) and its oxidized product 1-methyl-4-phenylpyridinium (MPP+). MPP+ selectively kills dopaminergic neurons in pars compacta of the substantia nigra, inducing parkinsonian symptoms in animals. Typically, neurotoxicity models of PD in zebrafish assess acute drug effects on locomotion. In the present study, we examined the lasting effects of MPP+ exposure and drug treatment in zebrafish larvae. Larvae were incubated in 500 μM MPP+, from 1 to 5 days post fertilization (dpf), followed by 24 h drug-free acclimation. At 6 dpf, the behavior was analyzed for locomotion, thigmotaxis, and sleep. Next, in separate assays we assessed the drug effects of brain injected glial cell-derived neurotrophic factor (GDNF) and 4-phenylbutyrate (PBA), co-incubated with MPP+. We show that MPP+ exposure consistently reduces swim distance, movement frequency, and cumulative time of movement; thus mimicking a parkinsonian phenotype of reduced movement. In contrast, MPP+ exposed larvae demonstrate reduced anxiety-like behavior and exhibit a sleep phenotype inconsistent with human PD: the larvae display longer sleep bouts, less sleep fragmentation, and more sleep. Previously reported rescuing effects of PBA were not replicated in this study. Moreover, whereas GDNF attenuated the sleep phenotype induced by MPP+, PBA augmented it. The current data suggest that MPP+ exposure generates a multifaceted phenotype in zebrafish and highlights that analyzing a narrow window of data can reveal effects that may be inconsistent with longer multi-parameter approaches. It further indicates that the model generally captures motor symptoms more faithfully than non-motor symptoms.

## Introduction

Parkinson’s disease (PD) is a neurodegenerative disorder with an estimated prevalence of 6.1 million individuals worldwide (Dorsey et al., 2018a). PD is the fastest growing neurological disease (Dorsey et al., 2018b) and the cause of progressive detrimental motor and non-motor symptoms. Motor symptoms result from a gradual degeneration of dopaminergic neurons within the substantia nigra pars compacta and subsequent striatal dopamine depletion (Maiti et al., 2017). Patients suffering from PD typically present one or more symptoms such as akinesia, bradykinesia, tremors, and rigidity, induced by alterations in neuronal activity (Moustafa et al., 2016). Gait disturbance, in particular, is one of the most commonly reported symptoms which manifests as impaired gait speed, increased movement variability, movement asymmetry, and reduced step length (Mirelman et al., 2019). Moreover, PD is associated with non-motor symptoms such as autonomic dysfunction, psychiatric disorders, and sleep disorders (Aygun, 2018). Autonomic dysfunction is commonly observed as cardiovascular symptoms such as hypertension or hypotension, or as gastrointestinal symptoms such as constipation, urinary complications, and sexual dysfunction (Asahina et al., 2013). Psychiatric disorders include anxiety, psychosis, apathy, impulse control disorder, Parkinson’s disease dementia, and depression (Han et al., 2018). Sleep disorders in PD are characterized as excessive daytime sleepiness, insomnia, fragmented sleep, or various REM movement disorders which can occur up to 10 years before the onset of motor symptoms (Pont-Sunyer et al., 2015).

Provisional treatment of PD-induced motor symptoms can be achieved with various types and combinations of drugs. PD has commonly been treated with the dopamine precursor levodopa, which is absorbed by the gastrointestinal tract and accesses the brain to induce elevated striatal dopamine levels (Koller and Rueda, 1998). However, after prolonged treatment with levodopa, the development of dopa-resistant motor symptoms and non-motor symptoms has been observed alongside levodopa-related side effects (Thanvi and Lo, 2004). Once levodopa turns ineffective or induces therapy-related motor complications, a set of other drugs can be prescribed such as dopamine agonists, catechol-o-methyl-transferase inhibitors, monoamine oxidase type B-inhibitors, and non-dopaminergic agents (Jankovic and Aguilar, 2008; Poewe et al., 2017). However, despite an increased understanding of the pathophysiology underlying PD and the continuous development of drugs providing symptomatic relief, a cure for PD is still lacking (Foltynie and Langston, 2018).

Several animal models of PD have been developed to aid the preclinical phase of drug discovery in the past decades. In 1983, 1-methyl-4-phenyl-1,2,3,6-tetrahydropyridine (MPTP) was linked directly to PD as several patients were hospitalized presenting with parkinsonian symptoms following self-administration of a drug containing the compound (Langston et al., 1983). The physical symptoms presented were, among others, generalized slowing and motor dysfunctions, and treatment with a combination of levodopa and carbidopa as well as dopamine agonist-therapy provided symptomatic relief, albeit no signs of remission. Increased interest in MPTP followed the discovery of MPTP-induced chronic parkinsonism, and it was subsequently used to establish animal models of PD (Langston, 2017).

Whereas MPTP itself is non-toxic, neurotoxicity occurs once metabolized by astrocytes to the neurotoxin 1-methyl-4-phenylpyridinium (MPP+; Ransom et al., 1987). MPP+ is actively taken up by dopamine transporter (DAT) and stored inside dopamine vesicles where it induces K^+^ efflux, cell membrane hyperpolarization, and inhibits neuronal firing. MPP+ ultimately inhibits complex I, decreases ATP production and promotes the generation of reactive oxygen species upon accumulation in the mitochondria (Yee et al., 2014). Both MPTP and MPP+ have been studied in several animal models of PD in the past years. MPTP has been assessed in mice (Jackson-Lewis and Przedborski, 2007), monkeys (Masilamoni and Smith, 2018), and zebrafish (Lam et al., 2005; Barnhill et al., 2020), where the reduction in dopamine cells is well documented (McKinley et al., 2005; Sallinen et al., 2009, 2010). Rats, however, show resistance to MPTP effects due to high activity of monoamine oxidases in the microvessels of the brain causing a rapid conversion of MPTP into MPP+, in combination with the inability of MPTP to pass the blood-brain barrier (Zeng et al., 2018). Locomotor perturbations in zebrafish larvae are observed following exposure to MPP+ as well as MPTP, although the latter has been shown to induce morphological alterations (Lam et al., 2005), cardiac defects, and tail curvature (Kalyn et al., 2019). A recent study, comparing differences in survival rate and morphology of zebrafish larvae following exposure to MPTP, MPP+, paraquat, 6-OHDA or rotenone, indicates that MPP+ is the optimal PD model as defined by dopaminergic ablation and mild locomotor perturbations while demonstrating the highest survival rate and absence of morphological changes (Kalyn et al., 2019).

It is evident that MPP+ exposure induces acute parkinsonian symptoms in zebrafish larvae and that various drugs provide acute symptomatic relief (Sallinen et al., 2009; Pinho et al., 2016). However, there is a gap in our knowledge of neurotoxic effects during extended behavioral recordings and the lasting effects of drugs that have proven effective in acute behavioral assays. Moreover, it is currently unknown to what degree the model recapitulates non-motor symptoms of PD. We have previously described the suitability of zebrafish for sleep-research (Sorribes et al., 2013; Srdanović et al., 2017), based on extended similarities to mammalian sleep. Furthermore, zebrafish possess highly conserved orthologues of human disease-associated genes and display human disease-related phenotypes (Howe et al., 2013). This establishes zebrafish as a valuable model for drug discovery (MacRae and Peterson, 2015; d’Amora and Giordani, 2018), with the advantages of low maintenance costs (Bilotta et al., 1999) and rapid development of transparent embryos, enabling researchers to visualize the development of vital organs and neuronal circuitry (Stewart et al., 2014). In relation to PD, the neuronal circuitry of zebrafish is well understood and the animal model mimics motor symptoms of PD in humans (Vaz et al., 2018). Thus, high-throughput screening (HTS) of candidate compounds can readily be performed in 96-well plates at the larval stage where compound permeability is high.

In this study, we revisited the MPP+ zebrafish model of PD with the intent to assess lasting (i.e., 24 h of drug-free acclimation followed by the 24 h recording period) effects of MPP+ exposure and establish a broad overview of behavioral parameters available for HTS in a 96-well plate format. To address the knowledge gap of the neurotoxic effects during extended behavioral recordings and lasting drug treatment effects, we assessed locomotion, thigmotaxis, and sleep phenotypes between 24 and 48 h after MPP+ exposure was ceased. Next, we examined the neurorestorative potential of glial cell-derived neurotrophic factor (GDNF) brain injected at 4 dpf, and the neuroprotective potential of 4-phenylbutyrate (PBA) co-incubated with MPP+. PBA has previously been shown to induce rescuing effects on motor symptoms in zebrafish larvae (Pinho et al., 2016), however, results from studies have been contrasting (Hegarty et al., 2016) and thus require further evaluation. Also, neurotrophic therapy using GDNF has been investigated as a treatment for PD by replenishing neurotrophic factors, and shown to exert both neuroprotective and neurorestorative effects (Allen et al., 2013) in rhesus monkeys (Gash et al., 1996), rats (Bohn et al., 2000) and rodents (Allen et al., 2013; Drinkut et al., 2016). The therapy has made it to clinical trials but did not reach its primary endpoints (Whone et al., 2019). Thus, further research is required to establish the response to GDNF treatment, and to our understanding, no such studies have been reported using zebrafish.

## Materials and Methods

### Zebrafish Husbandry

Wildtype zebrafish (AB line) were initially obtained from Oregon and have been maintained in the laboratory at the University of Reykjavik. Zebrafish were fed three times a day on a variable diet of TetraMin flakes (Tetra Holding GmbH, Melle, Germany), Adult Zebrafish Complete Diet (Zeigler Bros, Gardners, PA, USA), and live Artemia (INVE Aquaculture, Incorporation, Salt Lake City, UT, USA). Fish were kept in a 14:10 light:dark cycle (lights-on at 8:00 am) in 3 or 10 L multi-tank constant flow system tanks (Aquatic Habitats, Apopka, FL, USA). Water temperature was held at a constant of 28.5°C and replaced at a rate of 10% per day. Eggs were collected between 8:00–9:00 am and contained in 2 L tanks in system water (with 2 ml 0.1% methylene blue per 1 L). The following day, eggs were relocated to 92 × 16 mm Petri dishes (Sarstedt AG and Company KG, Nümbrecht, Germany) in groups of 40. For behavioral analysis, a total number of 415 larvae at 6 days post fertilization (dpf) were used in this study. All procedures in this study were carried out in strict compliance with the regulations of and approved by, the National Bioethics Committee of Iceland (regulation 460/2017).

### Drug Protocols

Zebrafish larvae were incubated between 1 and 5 dpf in the presence or absence of 500 μM MPP+ (Sigma–Aldrich, St. Louis, MO, USA) at a total volume of 25 ml system water. The drug solutions were freshly prepared and replaced between 11:00 and 12:00 am at 2, 3, and 4 dpf. Deceased larvae were removed daily, but no differences in mortality were observed between MPP+ exposed larvae and untreated larvae. Neuroregenerative effects of GDNF in MPP+ exposed larvae were assessed using human recombinant GDNF (Icosagen, Össu, Estonia) at a concentration of 8 ng/nl. The concentration was empirically determined based on unpublished data from previous experiments demonstrating low mortality and fast recovery rates. GDNF was administered as brain injections using a Drummond capillary (inner diameter 0.53 mm × outer diameter 1.14 mm) and Drummond Nanoinjector II (Drummond Scientific, BrookMall, Oakbrook, IL, USA) at 4 dpf. Capillaries were heated and pulled for a sharp tip (Narishige PC-10, Narishige, Japan) and inserted midline, post-ocular, and aimed at the tectal ventricle. Preliminary injections using dye were performed to verify the anatomical location and repeatability of injections. A total volume of 9.2 nl was injected, corresponding to 73.6 ng per larva. Before injection, larvae were sedated in 0.01% MS-222 (Sigma–Aldrich, St. Louis, MO, USA). After injection, larvae were briefly allowed to recover in system water before being incubated in 500 μM MPP+ for another 24 h. The effect of the pan-HDAC inhibitor, PBA (Sigma–Aldrich, St. Louis, MO, USA) was evaluated in the presence and absence of MPP+ by co-incubating the larvae between 1–5 dpf with 100 μM PBA. This concentration was chosen based on a previous publication in which rescuing of the locomotor pattern was demonstrated (Pinho et al., 2016). Both MPP+ and PBA were dissolved in the system water.

### Behavioral Recordings

At 5 dpf, larvae were placed in individual wells of 96-microwell plates (Nunc, Roskilde, Denmark) in system water. Thigmotaxis was recorded in 24-microwell plates. The microwell plates were relocated to a custom-built activity monitoring system fitted with 24 infrared cameras (Ikegami, ICD-49E; Ikegami Tsushinki Company, Japan) which was thermo-regulated at 28°C, blocked from daylight and illuminated from below with white (255 lx; light-phase) and infrared light (0 lx; dark-phase). Behavior was tracked in two dimensions at 5 Hz. Sleep parameters were calculated with a custom-written software. Larvae in all assays were left to acclimate in the activity monitoring system for 24 h before recording. The exclusion criterion was based on the percentage of samples during a recording where a larva was not tracked. The threshold was set to 10%, thus a larva that was tracked <90% of the total recording time was excluded.

### Motor Assay

Locomotor activity was recorded between 1:00 pm and 6:00 pm at 6 dpf during alternating light and dark conditions, presented in 30-min intervals. Four parameters were examined during five 30-min phases, following either light-to-dark transition or dark-to-light transition. Mean swim distance (mm) was calculated as the mean of the total distance swum during five separate 30-min time bins immediately after the transition of light conditions. Initiation of movement was defined as any instance of swim activity where velocity exceeded a threshold of 2 mm/s. Cessation of movement was defined as occurring when velocity dropped below a threshold of 1 mm/s. Movement frequency was defined as the total number of movements per 30-min time bin and represented as the mean of five separate time bins. The cumulative time of movement (s) was defined as the sum of time where a larva was moving during 30-min time bins. Mean cumulative time of movement was represented as the mean accumulated time spent moving during five separate time bins. Swim bout duration (s) was defined as the average duration per single swim bout and was calculated by dividing the movement frequency with the cumulative time of movement. Swim bout duration was represented as the mean swim bout duration during five separate time bins.

### Sleep Assay

Activity during sleep was recorded during the night period. The light was turned off at 10:00 pm and turned on at 8:00 am the next day. Sleep in zebrafish is defined solely using behavior. First, all behavior was dichotomized into 1-s bins of movement or non-movement. Prior, in-depth frame-by-frame video analysis by three independent raters resulted in the adoption of the speed of 1.0 mm/s as the threshold for movement for larval zebrafish. All activity that was slower than that threshold was computed as non-movement. Thus, rendering a dichotomized record of the behavior in either movement or non-movement. Next, the dichotomized record was transformed into bins of sleep and wake. Following previously established sleep criteria (Yokogawa et al., 2007; Sigurgeirsson et al., 2011; Sorribes et al., 2013), six or more consecutive 1-s bins of non-movement were counted as sleep and all else was counted as awake. That is, the seventh second and above were classified as sleep; all other bouts were classified as awake. Once the number of sleep and wake bouts was calculated, five different sleep parameters were assessed. Note that other labs have used a different time constant for adult and larval zebrafish (Rihel et al., 2010) however, stemming from work describing the ontogeny of sleep cycles in zebrafish, the same (short) time scales are used for larvae as adults (see Sorribes et al., 2013 for details). Sleep fragmentation was defined as the number of transitions between sleep and wake bouts per hour. The sleep ratio was calculated as the percentage of total night time that the fish was considered asleep. Sleep latency (s) was defined as the absolute time from lights-off until the first sleep bout. Wake bout duration (s) was defined as the average length of wake bouts. Sleep bout duration (s) was defined as the average length of sleep bouts.

### Thigmotaxis Assay

Thigmotaxis is an anxiety-like behavior characterized by avoidance of the arena center and a preference for the proximity of boundaries in a novel environment (Treit and Fundytus, 1988). Thigmotaxis in MPP+ exposed zebrafish larvae was assessed as previously described (Schnörr et al., 2012). Larval zebrafish were allocated individually into wells of a 24-well plate at 5 dpf and relocated to a recording room for 24 h of acclimation. Video recording was performed at 6 dpf and thigmotaxis was measured between 1:00 pm and 6:00 pm during alternating light conditions with white (255 lx; light-phase) and infrared light (0 lx; dark-phase) presented in 30-min intervals. An outer zone in each well was defined measuring 3 mm from the edge towards the center of the well. Thigmotaxis was calculated as the fraction of total movement performed in the outer zone during 30-min time bins following either light-to-dark transitions or dark-to-light transitions. Also, the distance from the center of the well was evaluated.

### Data Analysis

Data was obtained using EthoVision XT (Version 11.5.2016, Noldus) and exported to Microsoft Excel for analysis. Statistical analysis was performed using GraphPad Prism Software (Version 8.4.3, GraphPad Software Inc.). All data are presented as mean ± standard error of the mean (SEM). The D’Agostino and Pearson omnibus normality test was used to test for normality. For analysis of MPP+ exposed larvae and untreated larvae, a two-tailed unpaired *t*-test or two-tailed Mann–Whitney *U* test was used. For analysis of GDNF drug experiments, a two-tailed one-way ANOVA and Bonferroni *post hoc* analysis or two-tailed Kruskal–Wallis *U* test and Dunn’s *post hoc* analysis were used. For analysis of PBA drug experiments, a two-way ANOVA followed by Tukey’s *post hoc* analysis for multiple comparisons was used. *P* < 0.05 was considered statistically significant.

## Results

### MPP+ Exposure Reduced Swim Distance, Movement Frequency, and Cumulative Time of Movement During Lights-On

**Figure 1:**
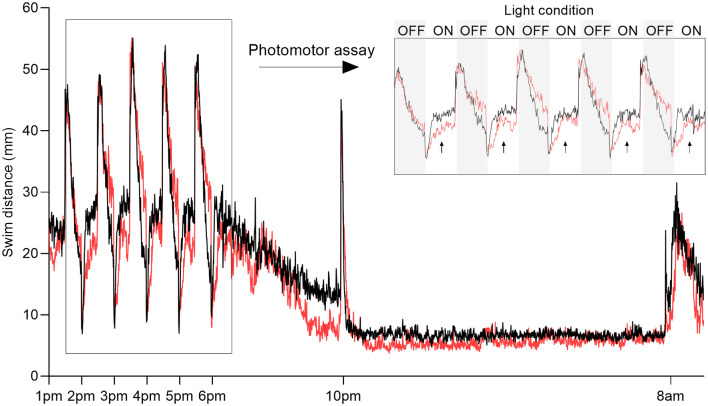
Overview of behavioral recording. Representative plot of swim distance of larvae exposed to 500 μM 1-methyl-4-phenylpyridinium (MPP+; red) vs. control (black). Inset (top right) shows the swim distance during photomotor assay measured between 1:00 pm and 6:00 pm consisting of five 30-min intervals alternating dark (OFF, gray boxes) and light (ON, white boxes). Arrows indicate the lights-on phases used for analysis. Sleep parameters were evaluated after the transition to darkness from 10:00 pm to 8:00 am the following day.

**Figure 2:**
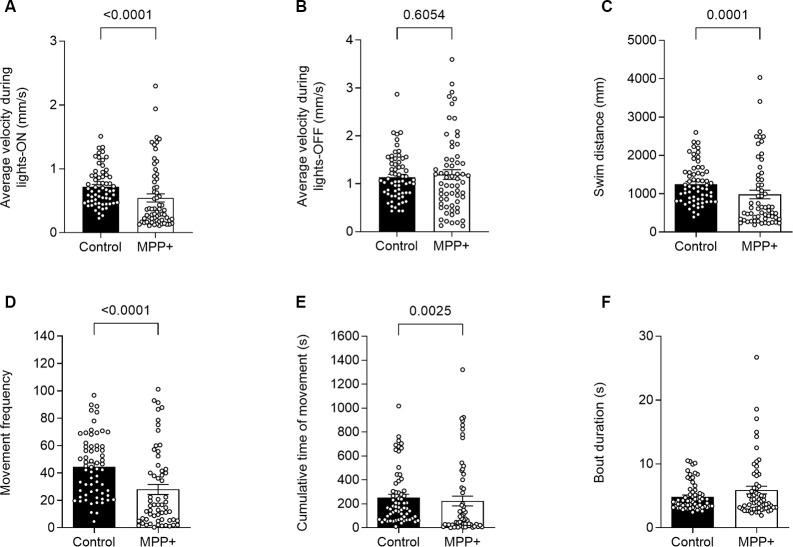
MPP+ effects on motor activity of zebrafish larvae. Motor activity was evaluated at 6 days post fertilization (dpf) in absence (Control) or presence of 500 μM MPP+ for **(A)** average velocity (mm/s) during lights-on and; **(B)** lights-off; **(C)** distance swam (mm); **(D)** frequency of movement; **(E)** cumulative time of movement (s) and **(F)** movement bout duration (s) were examined during five consecutive light-on phases. Data are presented as means ± SEM from two independent experiments pooled, control group *n* = 64, MPP+ group *n* = 61. Normality was tested using the D’Agostino and Pearson omnibus normality test followed by the nonparametric two-tailed Mann–Whitney *U* test.

To test the effect of the neurotoxin MPP+, known to target the DAT and mitochondria of dopaminergic neurons specifically, we exposed zebrafish larvae to 500 μM MPP+ and analyzed locomotion and photomotor response during alternating light and dark conditions in 30-min intervals (Figure 1). The effect of exposure to 500 μM MPP+ at the larval stage has previously been shown to mimic the effect of its precursor, MPTP (Lam et al., 2005). We observed alterations in locomotion of MPP+ exposed larvae during lights-on (Figure 2A), but not lights-off (Figure 2B). The average swim velocity of MPP+ exposed larvae was significantly lower during phases of lights-on (0.54 ± 0.063 mm/s vs. 0.72 ± 0.037 mm/s, *p* < 0.05) compared to untreated larvae. During phases of lights-on, MPP+ exposed larvae swam shorter distances (979.6 ± 113.19 mm vs. 1251.3 ± 65.38 mm, *p* = 0.0001) and showed a reduction in movement frequency (28.0 ± 3.68 vs. 44.6 ± 2.79, *p* < 0.0001) compared to untreated larvae (Figures 2C,D). Further, evaluation of swim bouts revealed that MPP+ exposed larvae spend significantly less cumulative time moving (223.6 ± 40.36 s vs. 252.5 ± 28.30 s, *p* = 0.0025) while maintaining similar swim bout durations (Figures 2E,F). These results demonstrate a phenotype characterized by fewer movement initiations leading to shorter swim distances as an effect of MPP+ exposure. Upon further evaluation of the cumulative time moving and duration of swim bouts, we show that MPP+ exposed zebrafish larvae spend less overall time moving compared to untreated larvae, but not due to shorter movement bouts. This indicates a parkinsonian phenotype in which movement is decreased.

### MPP+ Exposure Enhanced Sleep in Zebrafish Larvae

**Figure 3:**
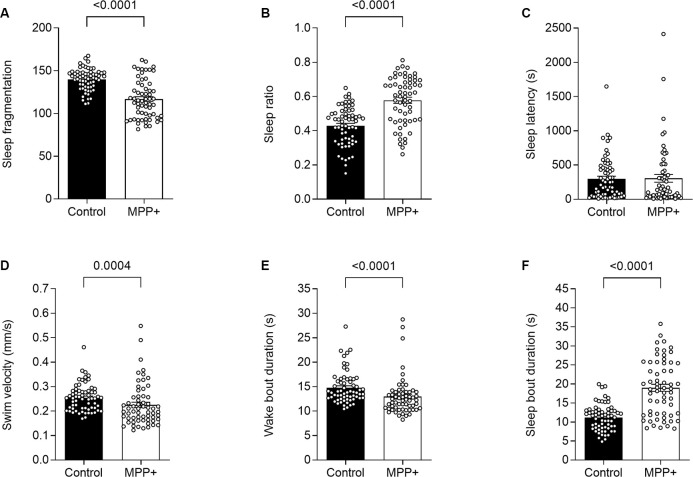
MPP+ effects on sleeping pattern of zebrafish larvae. Sleeping pattern was evaluated in absence (Control) or presence of 500 μM MPP+ for **(A)** sleep fragmentation; **(B)** sleep ratio; **(C)** sleep latency (s); **(D)** swim velocity (mm/s); **(E)** wake bout duration (s) and **(F)** sleep bout duration (s). Data are presented as means ± SEM from two independent experiments pooled, control group *n* = 64, MPP+ group *n* = 61. Normality was tested using D’Agostino and Pearson omnibus normality test followed by a two-tailed unpaired *t*-test or nonparametric two-tailed Mann–Whitney *U* test.

To our knowledge, the effect of MPP+ exposure on sleep phenotype in zebrafish larvae has not been previously reported. We analyzed locomotion and sleep-wake cycles during night from lights-off at 10:00 pm to lights-on at 8:00 am (Figure 1). Here, MPP+ exposed zebrafish larvae demonstrated a decrease in sleep fragmentation (117.2 ± 0.57 vs. 139.9 ± 0.43, *p* < 0.0001), swim velocity (0.23 ± 0.011 mm/s vs. 0.25 ± 0.0069 mm/s, *p* = 0.0004), and wake bout duration (13.0 ± 0.50 s vs. 14.8 ± 0.41 s, *p* < 0.0001) compared to untreated larvae (Figures 3A,D,E). In addition, MPP+ exposure increased sleep ratio (0.57 ± 0.018 vs. 0.43 ± 0.014, *p* < 0.0001) and sleep bout duration (18.8 ± 0.93 s vs. 11.2 ± 0.43 s, *p* < 0.0001) compared to untreated larvae (Figures 3B,F). There were no significant difference in sleep latency (Figure 3C). In agreement with our findings from the motor assay, MPP+ exposed larvae demonstrated less overall movement and an altered sleep pattern towards longer duration and less fragmentation.

### MPP+ Exposed Larvae Demonstrated a Reduced Anxiety-Like Behavior

**Figure 4:**
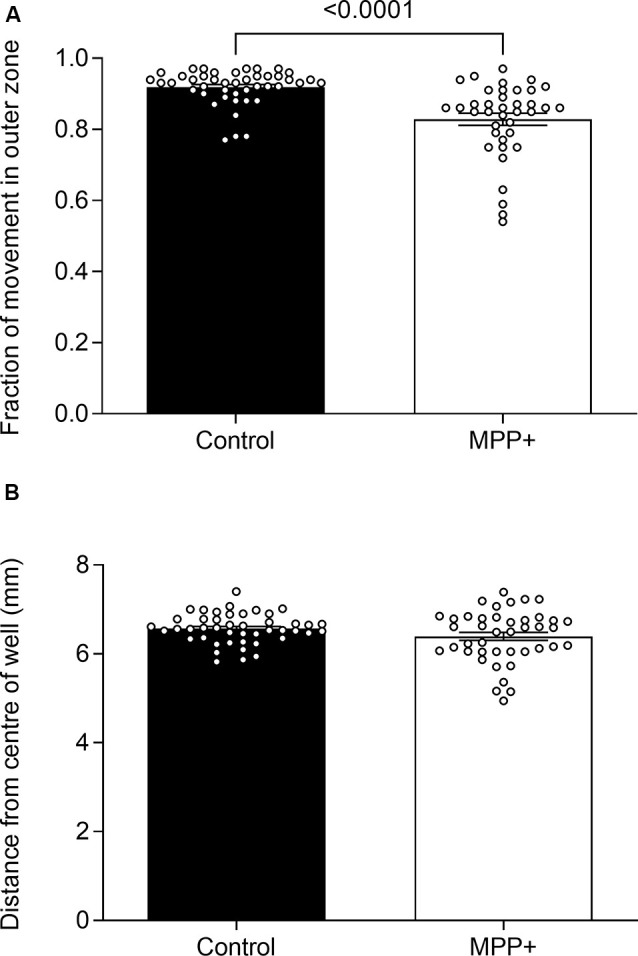
Effects of MPP+ on thigmotaxis. Thigmotaxis was evaluated at 6 dpf in the absence (Control) or presence of 500 μM MPP+ and represented as **(A)** the fraction of total distance swum in the outer zone of the well and **(B)** the average distance from the center of the well during lights-on. Data are represented as means ± SEM from four independent experiments pooled, control group *n* = 45, MPP+ group *n* = 42. Normality was tested using D’Agostino and Pearson omnibus normality test followed by a two-tailed unpaired *t*-test or two-tailed Mann–Whitney *U* test.

To study the effect of MPP+ exposure on the anxiety-like behavior of zebrafish larvae, we assessed thigmotaxis defined as a preference for dwelling in the periphery over the center of the well. To control for decreased absolute swim distances as an effect of MPP+ exposure, we assessed the fraction of the total swim distance performed in the outer zone during lights-on as opposed to absolute distances. We found that MPP+ exposure reduces thigmotaxis compared to untreated larvae (0.83 ± 0.0171 vs. 0.92 ± 0.00741 vs. *p* < 0.001; Figure 4A) while the mean distance to the center of the well was similar between groups (Figure 4B). These results indicate a less anxiety-like phenotype of MPP+ exposed larvae.

### GDNF Exerted No Rescuing Effects on the Locomotion of MPP+ Exposed Larvae, but Increased Movement and Decreases the Time of Sleep

**Figure 5:**
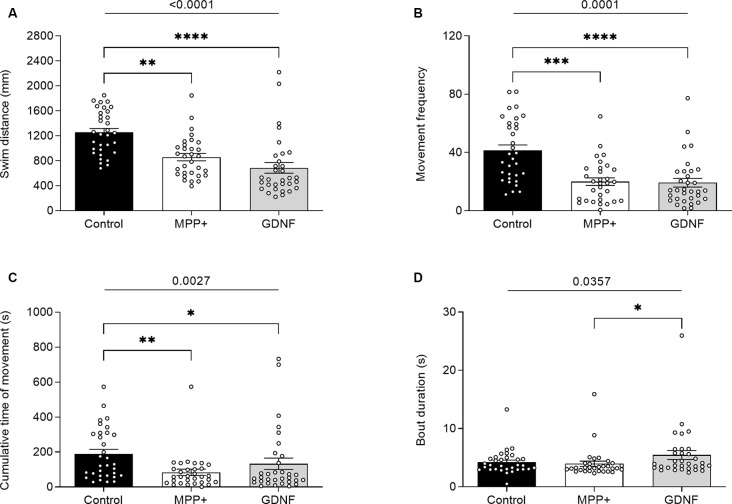
Effects of glial-derived neurotrophic factor (GDNF) on motor activity of MPP+-treated zebrafish larvae. Larvae were incubated in the absence (black bars) or presence (white and gray bars) of 500 μM MPP+ and injected with 72.9 ng GDNF (gray bars) at 4 dpf before assessing motor activity at 6 dpf for **(A)** distance swum (mm); **(B)** frequency of movement; **(C)** cumulative time of movement (s) and **(D)** swim bout duration during five consecutive light-on phases. Data are represented as means ± SEM from a single experiment, control group *n* = 32, MPP+ group *n* = 31, GDNF group *n* = 32. Normality was tested using D’Agostino and Pearson omnibus normality test followed by the two-tailed Kruskal–Wallis test (*p*-value shown above graph) and Dunn’s *post hoc* analysis. ****p < 0.0001, ***p < 0.001, **p < 0.01, *p < 0.05, for multiple comparisons.

**Figure 6:**
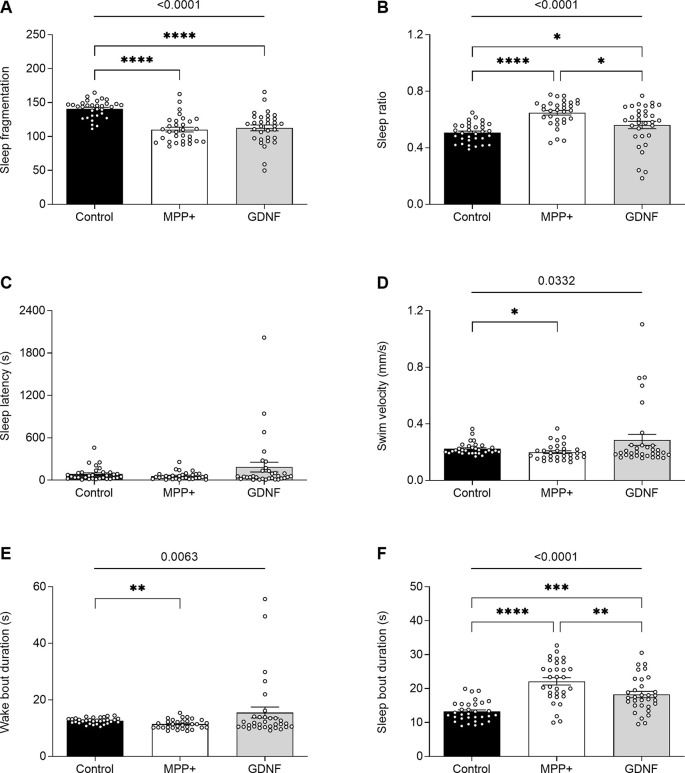
Effects of GDNF on the sleeping pattern of MPP+-treated zebrafish larvae. Larvae were incubated in the absence (black bars) or presence (white and gray bars) of 500 μM MPP+ and injected with 72.9 ng GDNF (gray bars) at 4 dpf before assessing **(A)** sleep fragmentation; **(B)** sleep ratio; **(C)** sleep latency (s); **(D)** swim velocity (mm/s); **(E)** wake bout duration (s) and **(F)** sleep bout duration (s) at 6 dpf. Data are presented as means ± SEM from a single experiment, control group *n* = 32, MPP+ group *n* = 31, GDNF group *n* = 32. Normality was tested using D’Agostino and Pearson omnibus normality test followed by the two-tailed Kruskal–Wallis test (*p*-value shown above graph) and Dunn’s *post hoc* analysis. ****p < 0.0001, ***p < 0.001, **p < 0.01, *p < 0.05, for multiple comparisons.

GDNF has previously been demonstrated to exert neuroregenerative effects in animal models (Allen et al., 2013). To test the potential of GDNF in a zebrafish model, we injected MPP+ exposed larvae with GDNF at 4 dpf. At 6 dpf, we observed a significant increase in swim bout duration for GDNF-injected larvae exposed to MPP+, compared to MPP+ exposed larvae alone (5.47 ± 0.77 s vs. 4.21 ± 0.37 s, *p* < 0.05; Figure 5D), but no difference in any other parameter (Figures 5A–C). Conversely, GDNF affected the sleep phenotype of zebrafish larvae by decreasing sleep ratio (0.56 ± 0.026 vs. 0.64 ± 0.016, *p* < 0.05) and sleep bout duration (18.3 ± 0.92 s vs. 22.1 ± 1.09 s, *p* < 0.01) compared to MPP+ exposed larvae (Figures 6B,F). No significant differences were found in any other sleep parameter (Figures 6A,C–E). This indicates an effect of GDNF considering that MPP+ exposure increases these parameters compared to untreated larvae. These results suggest that GDNF tempers the sleep phenotype induced by MPP+ and leads to increased movement during the night.

### PBA Failed to Rescue Locomotor Impairments Caused by MPP+ Exposure

**Figure 7:**
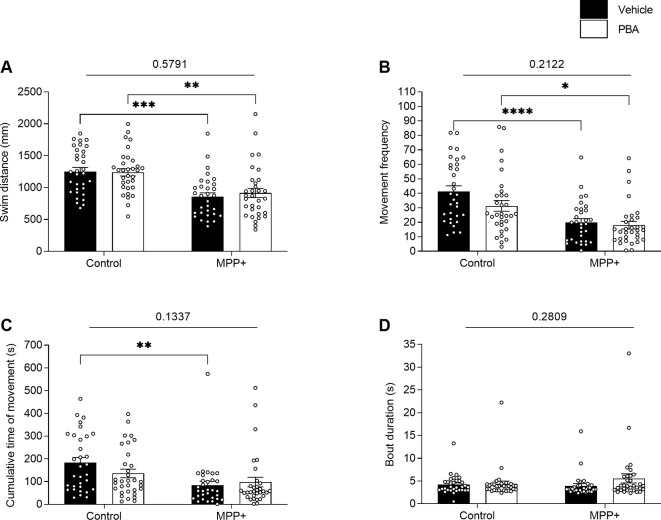
Effects of 4-phenylbutyrate (PBA) on motor activity of MPP+-treated zebrafish larvae. Larvae in the absence (Control) or presence of 500 μM MPP+ were treated with vehicle (black bars) or 100 μM PBA (white bars). Motor activity was evaluated for **(A)** distance swum (mm); **(B)** frequency of movement; **(C)** cumulative time of movement (s) and **(D)** movement bout duration (s) during five consecutive light-on phases. Data are presented as means ± SEM from a single experiment, control group *n* = 32, MPP+ group *n* = 31, PBA group *n* = 32, MPP+/PBA group *n* = 32. Normality was tested using D’Agostino and Pearson omnibus normality test followed by two-way ANOVA and Tukey’s *post hoc* analysis. *P*-value for interaction between neurotoxin exposure and drug treatment is shown above the graph. ****p < 0.0001, ***p < 0.001, **p < 0.01, *p < 0.05, for multiple comparisons.

PBA has previously been demonstrated to rescue acute locomotor impairments in zebrafish caused by MPP+ exposure following short durations of acclimation and behavioral recording (Pinho et al., 2016). To study the lasting effects of PBA on locomotion and sleep, zebrafish larvae were co-incubated with 100 μM PBA in the presence or absence of MPP+. Here, we did not observe any interaction effect between neurotoxin exposure and drug treatment. Consistent with previous findings, the two-way ANOVA reveals a significant main effect of exposure to MPP+ on swim distance (*F*_(1,123)_ = 32.02, *p* < 0.0001), movement frequency (*F*_(1,123)_ = 28.97, *p* < 0.0001) and cumulative movement time (*F*_(1,123)_ = 11.65, *p* = 0.0009; Figures 7A–C). *Post hoc* analysis shows a significant decrease in swim distance for larvae exposed to MPP+ compared to control larvae when treated with vehicle (*q* = 6.190, *df* = 123, *p* = 0.0001) and for larvae treated with PBA (*q* = 5.123, *df* = 123, *p* = 0.0024; Figure 7A). Similarly, a significant decrease in movement frequency was observed for larvae exposed to MPP+ compared to control larvae when treated with vehicle (*q* = 6.611, *df* = 123, *p* < 0.0001) and for larvae treated with PBA (*q* = 4.145, *df* = 123, *p* = 0.0207; Figure 7B). Cumulative time of movement was significantly reduced for larvae exposed to MPP+ compared to control larvae when treated with vehicle (*q* = 4.903, *df* = 123, *p* = 0.0040; Figure 7C). No significant difference was found in bout duration between groups (Figure 7D).

### PBA Induced a Sleeping Phenotype Independent of MPP+ Exposure

**Figure 8:**
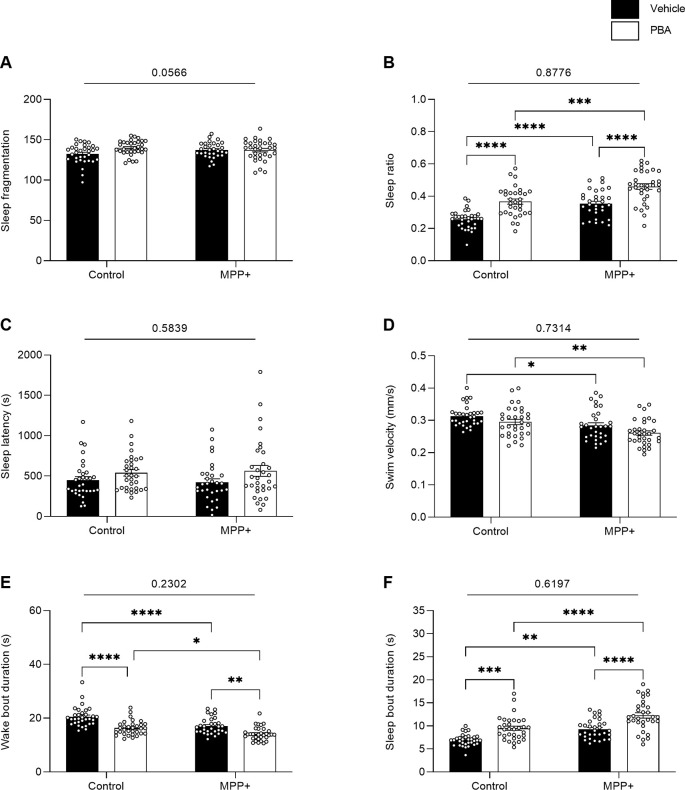
Effects of PBA on the sleeping pattern of MPP+ treated zebrafish larvae. Larvae in the absence (Control) or presence of 500 μM (MPP+) was treated with vehicle (black bars) or 100 μM PBA (white bars). Sleeping pattern were evaluated for **(A)** sleep fragmentation; **(B)** sleep ratio; **(C)** sleep latency (s); **(D)** swim velocity (mm/s); **(E)** wake bout duration (s) and **(F)** sleep bout duration (s). Data are represented as means ± SEM from a single experiment, control group *n* = 32, MPP+ group *n* = 31, PBA group *n* = 32, MPP+/PBA group *n* = 32. Normality was tested using D’Agostino and Pearson omnibus normality test followed by two-way ANOVA and Tukey’s *post hoc* analysis. *P*-value for interaction between neurotoxin exposure and drug treatment is shown above the graph. ****p < 0.0001, ***p < 0.001, **p < 0.01, *p < 0.05, for multiple comparisons.

The two-way ANOVA on sleeping parameters displays a significant main effect of exposure to MPP+ on sleep ratio (*F*_(1,123)_ = 40.89, *p* < 0.0001), swim velocity (*F*_(1,123)_ = 18.63, *p* < 0.0001), wake bout duration (*F*_(1,123)_ = 28.05, *p* < 0.0001) and sleep bout duration (*F*_(1,123)_ = 35.34, *p* < 0.0001; Figures 8B,D,E,F). Main effect of PBA treatment was observed for sleep ratio (*F*_(1,123)_ = 52.39, *p* < 0.0001), wake bout duration (*F*_(1,123)_ = 44.88, *p* < 0.0001) and sleep bout duration (*F*_(1,123)_ = 41.11, *p* < 0.0001; Figures 8B,E,F). *Post hoc* analysis highlights an increased sleep ratio for both MPP+ exposed larvae (*q* = 7.056, *df* = 123, *p* < 0.0001) and control larvae (*q* = 7.422, *df* = 123, *p* < 0.0001) when treated with PBA compared to vehicle (Figure 8B). Wake bout duration was significantly reduced for both MPP+ exposed larvae (*q* = 5.472, *df* = 123, *p* = 0.0010) and control larvae (*q* = 7.937, *df* = 123, *p* < 0.0001) when treated with PBA (Figure 8E). Conversely, sleep bout duration was significantly increased for MPP+ exposed larvae (*q* = 6.882, *df* = 123, *p* < 0.0001) and control larvae (*q* = 5.938, *df* = 123, *p* = 0.0003) following PBA treatment (Figure 8F). No significant differences in sleep fragmentation and sleep latency were found between groups (Figures 8A,C).

## Discussion

In alignment with reports on acute effects, we confirmed that MPP+ exposure induced mild locomotor deficiencies characterized by reduced swim distance, movement frequencies, and cumulative time spent moving. The change in velocity is robust and consistently observed during phases with lights-on in a photomotor assay with alternating light conditions, but does not appear during lights-off. Due to the absence of a direct parkinsonian gait, balance, and posture in zebrafish larvae, a further breakdown of movement by evaluating cumulative movement time and duration of swim bouts can provide a valuable tool that allows for a comparison to motor symptoms of human PD. As human PD progresses towards advanced stages, episodic gait disturbances are observed as festination, start hesitation, and freezing (Hausdorff, 2009). While hesitation to initiate movement can be addressed by monitoring movement frequencies alone, a combination of the cumulative time of movement and swim bout duration allows for a potential understanding of festination and freezing in zebrafish larvae. Whereas fewer movement initiations result in shorter swim distances, the decrease in cumulative movement time and unchanged movement bout duration indicates that MPP+ exposed larvae exhibits altered locomotion due to decreased movement frequency. Thus, the MPP+ model recapitulates the main hallmark of episodic gait disturbances in human PD patients by decreasing initiation of movement bouts leading to lesser overall movement. However, further research into the movement pattern of single movement bouts is needed to address whether festination and freezing similar to humans occur in zebrafish larvae.

Next, we assessed the effects of MPP+ exposure on sleep-wake cycles in zebrafish larvae during the night. In human PD patients, sleep impairments are often reported as increased sleep fragmentation, excessive daytime sleepiness, and REM behavior disorder (De Lazzari et al., 2018). We did not look into excessive daytime sleepiness and REM behavior disorder in the present study, but our sleep analysis allowed the characterization of sleep and wakefulness. We found that MPP+ exposed larvae display a phenotype inconsistent with the sleep phenotype observed in human PD patients. MPP+ exposed larvae present significantly lower sleep fragmentation, swim velocity, swim distance and wake bout duration, and, conversely, an increased sleep ratio and sleep bout duration. Previous work has described paradoxical sleep suppression in cats (Lin et al., 1995) and disruption of the molecular clock in mice (Hayashi et al., 2013) following exposure to the precursor of MPP+, MPTP, but to our knowledge, the effect of MPP+ exposure on sleep has not yet been investigated in animal models. These results suggest that the MPP+ model in zebrafish larvae does not recapitulate a sleep phenotype seen in human PD patients.

To elucidate whether MPP+ exposed zebrafish larvae exhibit an anxiety-like phenotype in alignment with non-motor symptoms of PD, we examined thigmotaxis characterized by avoidance of the arena center and a preference for the proximity to walls in a novel environment. Thigmotaxis has previously been validated in zebrafish larvae showing increased thigmotaxis following exposure to anxiogenic substances (Schnörr et al., 2012). Similarly, increased thigmotaxis has been observed in mice following a 6-OHDA lesion (Bonito-Oliva et al., 2014). Interestingly, our data revealed reduced thigmotaxis in MPP+ exposed zebrafish larvae as less swimming was performed in outer areas of the well. Thus, in the present study, MPP+ does not appear to enhance anxiety in zebrafish larvae questioning its application as a means to study non-motor symptoms of PD.

Previous research on the MPTP and MPP+ PD model using larval zebrafish has demonstrated rescuing effects of deprenyl (Sallinen et al., 2009), apomorphine, and 4-phenylbutyrate (PBA; Pinho et al., 2016). Deprenyl is a monoamine oxidase type B-inhibitor used to treat PD (Miklya, 2016). Sallinen et al. (2009) treated zebrafish larvae exposed to MPTP with 100 μM deprenyl at 0–4 dpf and reported a significant increase in distance moved, levels of dopamine, noradrenaline, and 5-hydroxytryptamine, as well as an increase in number of cells in four different dopaminergic populations compared to MPTP exposed zebrafish larvae alone. Pinho et al. (2016) assessed the effect of the dopaminergic agonist, apomorphine, exposing zebrafish larvae to 500 μM MPP+ at 3–5 dpf. They reported an increase in swim distance, swim velocity, circles performed, and movement heterogeneity compared to MPP+ exposed larvae alone. Similarly, following treatment with pan-histone deacetylase PBA, an increase in swim distance, time in movement, circles performed, and movement heterogeneity was observed compared to MPP+ exposed larvae alone. Both studies were performed using a brief acclimation period followed by a behavioral recording of spontaneous swimming of 10 min of duration. In the present study, we assessed the lasting effects of two compounds with different methods of delivery and different mechanisms of action. In addition to PBA, we chose GDNF based on the existing literature demonstrating neuroprotective and neurorestorative effects against exposure to MPP+ or MPTP both *in vitro* (Hou et al., 1996; Zeng et al., 2006; Zhang et al., 2008) and *in vivo* (Hoffer et al., 1994; Kojima et al., 1997). PBA was administered through co-incubation simultaneously with MPP+ exposure, whereas GDNF was injected into the brain at 4 dpf. Exposure to MPP+ and treatment with PBA was ceased at 5 dpf, followed by a 24 h acclimation period to the recording facility and recording at day 6 dpf. Following injection of GDNF, increased movement bout durations was observed whereas locomotion, in general, was similar to that of MPP+ exposed larvae. The effect of GDNF on sleep parameters was more pronounced as GDNF treated larvae showed a decrease in sleep ratio and sleep bout duration. These results suggest that GDNF enhances sleep of MPP+ exposed larvae and induces a sleep phenotype that is approaching untreated larvae. This is supported by existing literature showing that various growth factors such as GDNF are involved in sleep regulation (Kushikata et al., 2001). However, the absence of a sham group is a limitation and might have shed light on possible behavioral effects of the injection alone.

Following treatment with PBA, we replicated the phenotype of MPP+ exposed larvae observed during the initial motor assay and GDNF experiments. In contrast to our findings following GDNF injection, PBA on its own enhanced sleep characterized by increased sleep ratio and sleep bout duration, and conversely, decreased wake bout duration compared to control larvae independent of MPP+ exposure. Sleep-altering effects following PBA treatment are in agreement with a previous study in *Drosophila melanogaster* showing that acute administration of PBA causes aging flies to mimic a sleeping phenotype of young flies by modifying endoplasmic reticular stress (Brown et al., 2014). In contrast, these effects were not observed between groups of young flies. The key difference between this and previous studies is that drug exposure is ceased 24 h before recording. The effect of MPP+ is transient and in alignment with previous data (Sallinen et al., 2009), we have empirically determined that the effect is highest between 5–7 dpf and maximal at 6 dpf, following the incubation protocol applied in this study. Thus, examining lasting drug effects on MPP+ induced locomotor perturbations and sleep-wake cycles during extended recordings is a valuable addition to acute studies. These results confirm that, whereas mild locomotor perturbations induced by MPP+ persist following a 24 h acclimation period and an additional 24 h recording period, previously established acute drug effects are not recapitulated at this stage. Furthermore, while MPP+ exposed zebrafish larvae display a locomotor phenotype similar to human PD patients characterized by shorter swim distances and a lower movement frequency, the sleep phenotype does not match human PD patients. The putative transient nature of MPP+ could potentially explain this divergence. In the event that the effect of MPP+ exposure is most pronounced the first night, a lower effect during the second night of recording could potentially induce an overall increase in sleep in an attempt to recover. However, further studies are needed to assess sleep-wake cycles on consecutive nights as well as the potential role of excessive daytime sleepiness in zebrafish larvae.

In conclusion, we recapitulated mild locomotor perturbations as previously demonstrated in acute zebrafish models of PD following MPP+ exposure employing a 24 h acclimation period without drug exposure. We also show that neither PBA nor GDNF displayed rescuing potential at this stage. Also, we have demonstrated that MPP+ exposure does not induce an anxiety-like phenotype or a sleep phenotype similar to human PD patients. The current findings demonstrate that the MPP+ exposure model of PD in zebrafish generates a multifaceted phenotype that generally captures the motor symptoms of human PD more faithfully than non-motor ones.

## Data Availability Statement

The raw data supporting the conclusions of this article will be made available by the authors, without undue reservation.

## Ethics Statement

The animal study was reviewed and approved by and all procedures in this study were carried out in strict compliance with the regulations of, and approved by, the National Bioethics Committee of Iceland (regulation 460/2017).

## Author Contributions

CC, KK, and HÞ conceived and designed the study. CC performed the behavioral assays and injections, and also performed data analysis and data visualization. CC, VM, and KK wrote the manuscript. All authors contributed to the article and approved the submitted version.

## Conflict of Interest

KK and HÞ are shareholders in 3Z. The remaining authors declare that the research was conducted in the absence of any commercial or financial relationships that could be construed as a potential conflict of interest.
